# Unwanted incidents during transition of geriatric patients from hospital to home: a prospective observational study

**DOI:** 10.1186/1472-6963-10-1

**Published:** 2010-01-04

**Authors:** Marianne Mesteig, Jorunn L Helbostad, Olav Sletvold, Tove Røsstad, Ingvild Saltvedt

**Affiliations:** 1 Department of Clinical service, St. Olavs University Hospital, Olav Kyrres gt.17, 7006 Trondheim, Norway; 2 Department of Geriatrics, St. Olavs University Hospital, Olav Kyrres gt.17, 7006 Trondheim, Norway; 3 Department of Neuroscience, Norwegian University of Science and Technology (NTNU), Olav Kyrres gt.17, 7006 Trondheim, Norway; 4 Department of Health and Welfare, Municipality of Trondheim, 7004 Trondheim, Norway

## Abstract

**Background:**

Geriatric patients recently discharged from hospital experience increased chance of unplanned readmissions and admission to nursing homes. Several studies have shown that medication-related discrepancies are common. Few studies report unwanted incidents by other factors than medications. In 2002 an ambulatory team (AT) was established within the Department of Geriatrics, St. Olavs University Hospital HF, Trondheim, Norway. The AT monitored the transition of the patients from hospital to home and four weeks after discharge in order to reveal unwanted incidents.

The aim of the present study was to describe unwanted incidents registered by the AT among patients discharged from a geriatric evaluation and management unit (GEMU) by character, frequency and stage in the transitional process. Only unwanted incidents with a severity making contact with the primary health care (PHC) necessary were registered.

**Methods:**

A prospective observational study with patients treated in the GEMU and followed by the AT was performed. Current practice included comprehensive geriatric assessment and management including discharge planning in the GEMU and collaboration with the primary health care on appointments on assistance to be provided after discharge from hospital. Unwanted incidents severe enough to induce contact with the primary health care were registered during the transitional phase and after discharge.

**Results:**

118 patients (65% female), with mean age 83.2 ± 6.4 years participated. Median Barthel Index at discharge was 18 (interquartile range 16-19) and median Mini Mental Status Examination 24 (interquartile range 21-26). A total of 146 unwanted incidents were registered in 70 (59%) of the patients. Most frequent were unwanted incidents related to drug prescription regime (32%), exchange of information in and between the GEMU and the primary health care (25%) and service or help provided from the PHC (17%).

**Conclusions:**

Despite a seemingly well-organised system for transition of patients from the GEMU to their homes, one or more unwanted incidents occurred in most patients during discharge or four weeks post discharge. The study has revealed areas of importance for improving transitional care of geriatric patients.

## Background

Geriatric patients are often characterised as frail. Frailty is described as age-related physiological vulnerability, reduced homeostatic reserves and reduced capacity to withstand stress. It is associated with increased morbidity, functional decline, nursing home placement and death [1-3]. Frail elderly patients hospitalised for acute diseases are vulnerable for further functional deterioration after discharge [4] and a high frequency of unwanted incidents has been reported after discharge from hospital [4-12]. Several studies have shown that medication-related discrepancies or adverse effects of medications related to discontinuity are common [5-11], while few studies have focused on unwanted incidents by other factors than medications.

Continuity of care during transition from hospital to home and post-discharge follow-up is challenging. Several randomized controlled studies have shown positive effects of discharge planning and supporting patients over a limited period after discharge [13-29]. A time limited supported discharge in addition to treating geriatric patients in a specialized hospital unit has been shown to improve functional status [30-33] and increase possibilities of living at home [30], as well as to reduce mortality [4,30-33] and readmissions [31,33].

**Figure 1:**
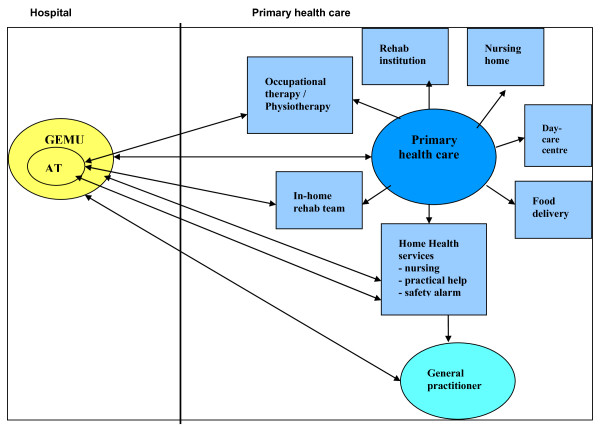
**Cooperation between the geriatric evaluation and management unit (GEMU) and the primary health care (PHC)**.

For many years St. Olav University Hospital has been collaborating with the primary health care (PHC) in the city of Trondheim, Norway in developing a system to improve the quality of transition of hospitalised patients to their homes [34-39] (Figure 1). As part of this work and based upon the literature referred above, an ambulatory team (AT) as part of the GEMU was established in 2002. The intention was to improve the transitional process from a GEMU to the patients' homes for patients living in Trondheim. The present study is aiming at describe unwanted incidents among patients discharged from the GEMU as part of such a practice by character, frequency and stage in the transitional process.

## Methods

### Study design

This is a prospective observational study. Patients were followed during the hospital stay and four weeks after discharge.

### Setting

Patients were recruited from the city of Trondheim which has about 160.000 inhabitants. The health care system of Norway is public with hospitals financed by the government, while the primary health care including the nursing homes are financed by the municipalities. Assistance from home care nurses is free, but patients are charged for practical help in their home according to their income.

The study was run from a 15-bed GEMU as part of the Clinic of Internal Medicine at St. Olav University Hospital in Trondheim, Norway, the study period lasting from November 2005 throughout June 2007. Approximately 80% of the admitted patients lived in their own homes and were referred to the hospital as emergencies caused by acute medical diseases.

### Sample

All patients planned to be discharged from the GEMU to their homes and who got supported discharge from the AT was asked to participate. Patients being discharged to nursing homes, rehabilitation institutions or other hospital departments were not included. For practical reasons AT services were only offered to patients living in the city of Trondheim. Hence, patients from surrounding municipalities were not included.

### Follow-up of patients from the GEMU to the primary health care

#### In hospital

The GEMU patients went through a comprehensive geriatric assessment (CGA) by an interdisciplinary team consisting of physicians, nurses, occupational therapists, physiotherapists and registered nurses. Discharge planning was emphasised and started as early as possible. More details about the CGA are described in a previous publication [37].

A discharge-planning meeting was arranged for all GEMU-patients during the hospital stay. Participating in this meeting were the patient, his closest caregiver(s), the attending physician, nurse and the AT primary contact in the GEMU and participants from the primary health care. During the meeting the primary health care received information on the patient's medical and functional status as assessed in the GEMU and assistance and adjustments necessary after discharge were discussed. At the end of the meeting the primary health care representative wrote a detailed decision on services to be provided by the primary health care after discharge, specifying assistance related to activities of daily living (ADL) and instrumental activities of daily living (IADL) including administration of drugs, services in a day-care centre, inpatient or ambulatory rehabilitation, short- or a long-term stay in nursing homes, physiotherapy, occupational therapy, in-home rehabilitation, food delivery and acquisition of safety-alarms. After this meeting the primary health care representative reported the patient's status and assistance to be provided to the office of home health services (HS) (Figure 1). The service should be performed according to the primary health care's standards, including method and time to be used for the different services provided in the patient's home [40]. Sometimes, the discharge planning meeting was arranged in the patient's own home. In these cases a representative from the home services who knew the patient beforehand was present (in addition to the representative from the primary health care), and only the patient's AT primary contact participated from the GEMU.

Members of the AT were of a nurse, an occupational therapist and a physiotherapist, all being experienced with specialised education and training in geriatrics. The physicians in the GEMU served as medical consultants for the AT. The intention of the follow-up by the AT was to cooperate with the primary health care and the GEMU in order to prevent, uncover and resolve unwanted incidents of importance for the patient's situation, e.g. misunderstandings concerning medications, absence of assistance from the primary health care or problems influencing the patients' health condition after discharge.

#### Transition

At discharge the attending physician in the GEMU wrote a discharge letter to the patient including information on diagnosis, drug regimen and medical follow-up after discharge, and a copy was sent to the home care nurse. A complete medical report was later sent to the patient's general practitioner. In addition, the GEMU team members sent a case summary to the responsible carer in the primary health care with detailed recommendations on follow-up of e.g. nutrition, physical exercise, adjustments of home environment.

#### After discharge

After discharge the patients were expected to receive assistance from the primary health care as decided during the stay in the GEMU according to the routines of the home health services. The AT visited the patients during the first week after discharge, while contact afterwards was generally maintained by telephone calls to the patient, the closest caregiver, or representatives from the primary health care, at least once a week during a follow-up period of four weeks. Occasionally representatives from the primary health care contacted the AT. Figure 1 gives a schematic outline of the clinical practice regarding the cooperation between the GEMU and the primary health care.

### Procedures

Baseline characteristics were collected from the patient's hospital record, the hospital database and the decision from the discharge-planning meeting.

During the first four weeks after discharge the AT team members, registered predefined unwanted incidents on registration forms. Only unwanted incidents with a severity making contact with the primary health care necessary were registered. If uncertainty whether or not it should be defined as an unwanted incident, a consensus was achieved with the project coordinator, the senior medical officer of geriatric medicine in Trondheim and with an experienced geriatric nurse in the GEMU.

### Outcome variables

Cognitive function was assessed during the hospital stay by an occupational therapist, using the Mini Mental State Examination (MMSE) [41]. ADL was assessed by the Barthel Index (BI) [42] at admission and discharge by other GEMU personnel.

**Figure 2:**
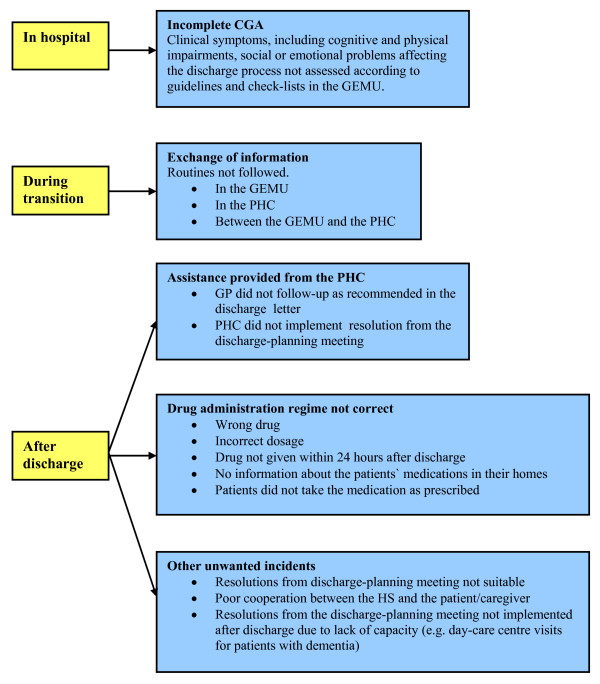
**Endpoints evaluated in the present study as unwanted incidents**. CGA = comprehensive geriatric assessment; GEMU = geriatric evaluation and management unit; PHC = primary health care; GP = general practitioner; HCN = home care nursing; AT = ambulatory team; HS = home service.

The GEMU staff members, representatives for the AT as well as the primary health care participated in the planning of the study. Main outcomes of this study were unwanted incidents registered during the hospital stay, at discharge and during four weeks of follow-up. Unwanted incidents to be registered were defined according to a literature review and clinical experience with 230 patients during a running-in period of two and a half year. A standardized instrument was developed, tested out in a pilot study, revised and then implemented as the final registration form. The form was designed to register a diversity of incidents related to the transitional process from hospital to the patients' homes. The unwanted incidents were detected through contact with patients and caregivers and observations made at home visits by the AT. Figure 2 describes study endpoints.

Criteria for registering unwanted incidents were 1) the CGA during the stay in the GEMU turned out to be incomplete, 2) routines for exchange of information between the GEMU and the primary health care were not followed, 3) the drug regimen from the GEMU was not administrated correctly by the home services or the patient, 4) discrepancies in the decision from the discharge-planning meeting and the assistance actually provided by the primary health care, or 5) the guidelines for practical assistance in the patient's homes were not followed.

### Statistical analysis

Statistical analyses were performed in SPSS version 14.0. An alpha level of 0.05 was chosen for statistical significance. Differences between included and non-included patients were tested by Independent Samples t-tests for ratio data and Pearson's Chi-Square tests for nominal data. The Wilcoxon signed ranks test was used to test change in Barthel Index score from admission to the GEMU till discharge. Binary logistic regression analysis was used to test whether length of stay, the patient living alone or not, the amount of assistance from the HS and Barthel Index score at discharge, explained the probability of an incident to occur.

### Ethics

Participation was voluntary and in accordance to the Helsinki declaration. Those who refused to participate received the same treatment and follow-up from the GEMU, AT and PHC as participating patients. All patients received both oral and written information about the project. The Regional Committee for Ethics in Medical Research and The Norwegian Social Science Data Services approved the protocol.

## Results

During the study period 170 patients being discharged to their homes were asked to participate, of whom 39 refused, giving a study sample size of 131 patients. Moreover, data from 13 of the enrolled patients' were excluded from analyses because main outcome variables had not been registered. Thus, the analyses involve 118 patients (69.4% of those who were invited to participate). At baseline there were no significant differences in proportion of women (p > 0.3), age (p > 0.8) or independent living (p > 0.6) between enrolled patients and patients refusing to participate, or between patients included and excluded from the analysis (gender p > 0.9, age p > 0.2 and independent living p > 0.7, respectively).

Patient characteristics are described in Table 1. Mean age was 83.2 (SD = 6.4) years, 65.3% were women, and 65.3% lived alone. The median length of stay was 9 days (interquartile range (IQR) = 7-13). Table 2 describes assistance provided by the primary health care. One-hundred-and-four (88%) of the 118 patients received assistance from the home services after discharge. Seventy-four (62.7%) patients had more than one visit per day, 19 (16.1%) had one visit per day, and 11 (9.3%) had one visit per week. Most common was assistance related to house cleaning, drug regimen and shower/bath once a week. As shown in Table 2, 20 - 30% underwent some kind of therapy (physiotherapy, occupational therapy, in-home rehabilitation) after discharge, while, 45% had weekly visits in a day care centre and 81% had a safety alarm.

**Table 1:** Baseline characteristics (n = 118)

Characteristics	
Age, years; mean (SD/range)	83.2 (± 6.4/66-98)
Females; n (%)	77 (65.3)
Living alone; n (%)	77 (65.3)
Hospital length of stay, days; median (IQR)	9 (7-13)
Discharge-planning meeting in the GEMU; n (%)	51 (43.2)
BI score at admission (n = 86), 0-20; median (IQR)	17 (13-19)
BI at discharge (n = 86), 0-20; median (IQR)	18 (16-19)
MMSE during the hospital stay (n = 77) 0-30; median (IQR)	24 (21-26)

SD = standard deviation; IQR = interquartile range; GEMU = geriatric evaluation and management unit;

BI = Barthel Index scale; MMSE = Mini Mental State Examination

**Table 2:** Assistance provided by the primary health care (n = 118)

	n (%)
*Home Services*	104 (88.1)
Cleaning	47 (65.3)
Drug regimen	74 (62.7)
Help with shower/bath once a week	65 (55.1)
Morning hygiene	38 (32.2)
Food/eating	42 (35.6)
Visit to check the patient's status	31 (26.3)
Other	26 (22.0)
*Therapy*	
Physiotherapy	31 (26.3)
Occupational therapy	15 (12.7)
Rehabilitation in-home team	24 (20.3)
*Institution*	
Day care centre for elderly people	53 (44.9)
Rehabilitation centre stay	13 (11.0)
Short-time nursing home stay	9 (7.6)
*Food delivery service*	29 (24.6)
*Safety alarm*	96 (81.4)

### Frequency and character of unwanted incidents

**Figure 3:**
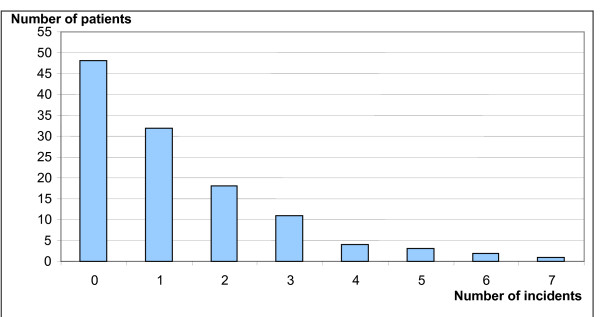
**Total numbers of unwanted incidents experienced per patient (n = 118)**.

A total of 146 unwanted incidents were registered in 70 (59%) of the patients, giving a median of 2 incidents per patient. Figure 3 shows the total number of unwanted incidents experienced per patient. Binary logistic regression analyses showed that incidence occurrence was higher among patients who received assistance from the home services several times a day as compared to patients receiving assistance once per week or once per day (p = 0.02).

**Figure 4:**
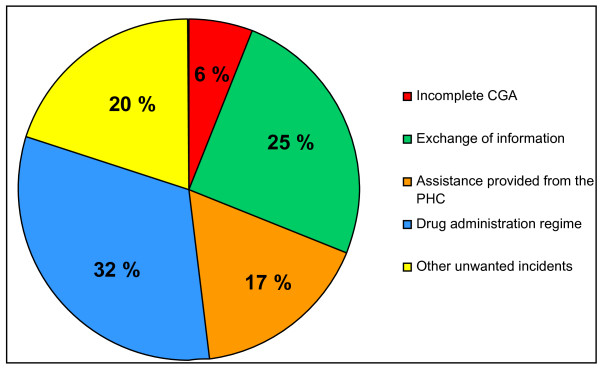
**Distribution of unwanted incidents**.

The most commonly reported incidents were related to 1) drug prescription regime, exemplified by the home services not replacing the patients' old list of medications with a new one after the hospital stay, or that the patient got the wrong drug or incorrect dosage, 2) exchange of information, e.g. patients were not referred to a specialist as planned before discharge, messages were not passed on, there were shortcomings in the medical report from the attending physician or in case summaries from the nurse, or decisions about the patient's assistance decided in the discharge-planning meeting were not passed on from the representative from the primary health care to the home services, 3) service or help provided from the primary health care, e.g. they did not make sure that the patients ate their meals (as the decision said they should) and 4) decisions made in the discharge-planning meeting in the GEMU were not relevant for the patient after discharge, e.g. the patient needed assistance with the prescription regime after all. The distribution of the unwanted incidents is shown in Table 3, and the percent-wise distributions of the incidents are shown in Figure 4.

**Table 3:** Distribution of unwanted incidents (N = 118)

Distribution	Number of patients experiencing unwanted incidents (N = 118)	Number of unwanted incidents (N = 146)
	***n (%)***	***n (%)***

***Incomplete CGA***	9 (7.6)	9 (6.2)

Emotional problems		1

Cognitive impairments		3

Physical impairments		1

Clinical problems		3

Social problems		1

***Exchange of information***	27 (22.9)	36 (24.7)

In the GEMU		8

In the PHC		13

Between the GEMU and the PHC		15

***Drug administration regime***	36 (30.5)	46 (31.5)

In the PHC		

• Wrong drug/incorrect dosage		16

• Drug not given within 24 hours after discharge		8

• No written information on drug regimen in patients' homes		8

• Medication not taken by patient as prescribed		10

In the GEMU		4

***Assistance provided from the PHC***	23 (19.5)	25 (17.1)

Patient not controlled by GP as recommended by the GEMU		5

Disagreement between services appointed and services offered		20

***Other unwanted incidents***	23 (19.5)	29 (19.9)

Resolution from discharge-planning meeting not suitable		11

Poor cooperation between HS and patient/caregiver		14

Not possible to implement resolution from discharge-planning meeting		4

CGA = comprehensive geriatric assessment; GEMU = geriatric evaluation and management unit; PHC = primary health care; GP = general practitioner; HS = home services

During the follow-up period three of the patients needed more help from the HS, five patients visited the GP in his office and another five were visited by the GP in their home, there were six unplanned readmissions to the hospital and one admission to a nursing home and one patient died.

## Discussion

Clinical experience and previous studies have shown that unwanted incidents after discharge of patients from hospital to their homes are common, especially related to discrepancies in drug regimen [4-12]. The present study has investigated the frequency and character of unwanted incidents observed by the AT during the transition of frail elderly patients from a geriatric hospital ward to their homes. It was shown that nearly 60% of the patients experienced unwanted incidents during transition and during the first four weeks after discharge. The most frequent incidents were mismatches between assistance appointed and assistance actually provided by the primary health care, errors regarding drug regimen and errors related to exchange of information.

Despite specific guidelines and accepted routines for transition from hospital to home, most patients in the present study experienced one or more unwanted incidents. This may have multiple explanations. Firstly, the study group was frail, of high age, often living alone, and having disabilities, including cognitive impairment, making them dependent of assistance in many daily life situations and vulnerable for experiencing incidents.

Secondly, although both the GEMU and the primary health care continuously work on improving communication within and between the systems, the large number of helpers around the patient requires that information about the patient is exchanged between different professions and employees several times and thereby increase the likelihood of unwanted incidents.

Thirdly, the skills and focus of the GEMU and the primary health care staff were different. The interdisciplinary staff of the GEMU emphasises optimal treatment for disorders precipitating somatic and psychiatric conditions, functional problems and the social situation of relevance for the actual hospital stay, while the main focus for the primary health care is to make it practicable and possible for the patient to stay at home as long as possible. Furthermore, the GEMU staffs was specialized in geriatric medicine while most of the employees in the primary health care had no specialised training in treating the actual patient group, many were unskilled. This may to some extent also explain why the patients did not receive help that was specifically appointed by the primary health care (Table 3).

However, the study also revealed that some decisions made in the discharge-planning meeting in the GEMU were not relevant after discharge (Table 3). Such errors were not registered for discharge-planning meetings arranged in the patients' home where also a representative from the home services who knew the patient beforehand was often present, demonstrating that the context of this meeting is important.

In concordance with other studies [5-11] we found unwanted incidents related to drug regimen to occur frequently. We did not evaluate whether these errors constituted a risk for the patients' health, but according to clinical experience this could be the case in some occasions, while in other situations these errors probably had no immediate impact on health status.

As shown in Figure 1, GPs are not routinely involved in follow-up of the patient during or shortly after discharge from our hospital. Hansen et al. have shown that patients recently discharged from hospital are not medically stabilised [11]. Their study uncovered problems during the first 3 weeks after discharge resulting in changes of medical or social treatment plans in the majority of patients. To further improve practice for these frail patients, a closer follow-up from the general practitioner shortly after discharge from hospital need to be highlighted. The length of hospital stay is constantly squeezed down, making the involvement of the general practitioner increasingly important.

It was shown that the patients in the present study during follow-up needed consultations from GPs, new hospital and nursing home admissions and one patient even died. However, the study was not designed to evaluate if these events were consequences of the unwanted incidents registered and thus could be prevented. In a controlled study it would be possible to evaluate if improvement in the transition from hospital to home could improve patient related outcomes.

Studies have shown that post-discharge visits in the patient's home by competent professionals can reveal important and potentially reversible clinical problems and unwanted incidents [13-29] and there are indications that functional status improves after a time-limited supported discharge [26,30-33]. The large number of incidents registered in the study could indicate that the AT in general should be more active in the management of the patients, such as following the patient home at discharge to make sure that assistance take place as appointed [43], perform treatment in the patients homes that the home services are not competent to provide [31,34], or working closely with the general practitioner during the transition to avoid medical incidents [31].

Strength of our study was the comprehensive registration of a diversity of incidents related to the transitional process from hospital to the patients' homes and not only disagreements related to drug regimens. Most likely this has resulted in a higher number of incidents than registered in earlier studies. We argue that this broad perspective has given important knowledge about aspects of the patients' situation to further improved discharge planning and follow-up. Though, it is still not known, though to what extent the registered incidents in our study influence the patients' and the caregivers' quality of life and function, hospital readmissions, nursing home placement or death. This needs to be highlighted in future studies.

The study has some limitations. Firstly, the practice for the cooperation between GEMU, AT and the primary health care described in the present study is unique for the city of Trondheim and the external validity may be questioned. However, we believe that the challenges observed are recognisable also for frail patients in general when focusing on transitional care. Secondly, we were not able to find standardised questionnaires for registration of relevant unwanted incidents. Therefore an incidence form was developed and revised through a pilot study; however it was not tested for reliability. Filling in the form was based on consensus between the GEMU, the AT and the primary health care which should reduce the possibility for systematic bias. Thirdly, according to the pragmatic design of the study, the AT had a double role being involved both in the planning of the transitional care during the hospital stay, but also in uncovering and resolving unwanted incidents of importance for the patient's situation, as well as registering unwanted incidents. This might have reduced the number of incidents, indicating that the incidence rate could have been even higher than registered without an AT.

## Conclusions

Despite a seemingly well-organised system for cooperation between the GEMU and the primary health care, unwanted incidents occurred in approximately 60 percent of frail elderly patients during transition from hospital to home and four weeks of follow-up. The majority of unwanted incidents were related to exchange of information, drug regimens and disagreements between services appointed and services provided by the primary health care. The study both demonstrate the need for and the challenges in designing a well-functioning system for this complex patient group both during the hospital stay as well for the time after discharge. The knowledge gained through the study has given insights of importance for future improvements of transitional care for geriatric patients.

Further studies, including interventional studies and evaluation in other settings, are necessary to investigate if these results are valid outside Trondheim and if improvements have impact on patient related outcomes such as quality of life, unplanned readmissions and nursing home placement.

## Competing interests

The authors declare that they have no competing interests.

## Authors' contributions

MM, OS, and IS planned the study. MM and TR took part in data collection. MM and JLH have analyzed the data and MM has written the paper in close cooperation with the other authors. All authors contributed to the development and revision of the manuscript and approved the final version.

## Pre-publication history

The pre-publication history for this paper can be accessed here:

http://www.biomedcentral.com/1472-6963/10/1/prepub
